# Effect of 3D Printed Spatial Reinforcement on Flexural Characteristics of Conventional Mortar

**DOI:** 10.3390/ma13143133

**Published:** 2020-07-14

**Authors:** Jacek Katzer, Tomasz Szatkiewicz

**Affiliations:** 1 Faculty of Geoengineering, University of Warmia and Mazury in Olsztyn, 10-720 Olsztyn, Poland; 2 Faculty of Mechanical Engineering, Koszalin University of Technology, 75-620 Koszalin, Poland; tomasz.szatkiewicz@tu.koszalin.pl

**Keywords:** 3D printing, spatial reinforcement, quasi-plastic, concrete, steel substitution

## Abstract

In their fourth decade of development, additive manufacturing technologies are slowly entering research programs dedicated to building materials. While the majority of research effort is focused on using 3D printing of concrete, the authors propose using the technology for creation of spatial plastic reinforcement. Obviously, the strength properties of a 3D printed polymer are much lower than those of steel. Nevertheless, the unconventional spatial shape of a 3D printed reinforcement can substitute for much of the lower mechanical performance of polymer. Flexural characteristics of a cement mortar prism specimen reinforced by hexagon spatial elements were tested and analyzed in this paper. The hexagonal geometric shape was chosen due to its high rigidness. It was proven that it is possible to efficiently reinforce concrete beams by spatial 3D printed polymer elements. Directions of needed research were pointed and discussed.

## 1. Introduction

The term additive manufacturing [1,2] is used to describe the technologies that build 3D objects by adding layer upon layer of a material. Currently, 3D printing is the best known and globally disseminated additive manufacturing technology. It is based on digitally controlled processes in which material is joined (or sometimes solidified) to create a new object. Different types of polymers remain the most popular materials used for 3D printing, but metals and concrete are recently gaining substantial popularity. The latter caught the attention of research teams dealing with building materials and civil engineering [3,4]. All over the world, multiple scientific institutions are currently involved in research programs dedicated to 3D printing of concrete, mortar [5] or concrete-like composites based on extra-terrestrial soil simulants (mainly lunar and Martian) [6,7,8]. The achieved development of the technology enabled the creation of geometrically complicated curved walls (both solid and hollow) and full-scale demonstrators. Nevertheless, key problems with bonding between printed layers of concrete and lack of reinforcement remain intact and significantly limit its potential for civil engineering applications [9,10,11].

All processes associated with production, transportation, casting and compacting (if needed) of concrete are highly automated. 3D printing of concrete and mortars (including fiber-reinforced mortars [12]) seems, in this context, like over-engineering of optimized and well-working procedures. On the other hand, preparation and placement of steel reinforcement is usually the most complicated and time-consuming activity at a construction site [13]. Reinforcing steel is expensive and characterized by a large carbon footprint [14]. The very process of reinforcement preparation, consisting of steel bars, stirrups, meshes and other smaller elements, is prone to human errors [15]. In the authors’ opinion, 3D printing in civil engineering should be primarily harnessed for the creation of reinforcement. The most laborious process in the erection of a concrete structure would be finally fully automated. One could also argue that using steel as a reinforcement is very conservative and was justified when no other technological solutions were available. 3D printing should be recognized as a disruptive technology and used to its limits [16]. The development of traditional steel reinforcement reached its technological limits with the production of fiber used for the creation of steel fiber-reinforced concrete (SFRC) [17] and slurry-reinforced concrete (SIFCON) [18]. Technology of traditional production and casting of concrete should be merged with 3D printing of polymer reinforcement. Mechanical properties of any 3D printed polymer are significantly lower than characteristics of any steel (especially in terms of strength and modulus of elasticity). In the authors’ opinion, this obvious weakness of 3D printed polymer reinforcement can be substituted by creation of a very complicated spatial shape with artificially created roughness of its surfaces. Such spatial reinforcement, creation of which is not possible using rebars, would match the strength properties of steel cages. The mechanical effectiveness of traditional steel rebars is obstructed by possible to achieve (by bending, cutting and welding) spacing in a concrete element. In contrast, 3D printed polymer reinforcement is almost limitless in the case of its geometry and spacing (e.g., 3D printed formwork playing the role of reinforcement). This concept was proved by authors during the previous research program dedicated to 3D printed polymer reinforcement which played a role of traditional reinforcement [19]. The success of the previous research program encouraged authors to prepare a new one described in this paper. During the realization of the research program, different spatial reinforcing elements were created based on hexagonal geometry [20]. The hexagonal geometry was chosen for the spatial reinforcement due to its proven rigid mechanical characteristics [21]. It was also successfully used for creation of beam-like lattice structures [22]. Spatial reinforcing elements were used to reinforce cement mortar beams. The main testing focus was dedicated to flexural strength of the beams and their load–deflection characteristics. All specimens with 3D printed polymer reinforcement were characterized by quasi-plastic properties.

## 2. Materials and Methods

Standardized mortar commonly used for tests of cements (EN 196-1) [23] was adopted as a brittle matrix. For the creation of the mortar CEM I 42.5 N-NA, standardized CEN sand (EN 196-1) characterized by median diameter [24] of 0.24 mm and tap water were used. The mixture composition of the mortar is given in Table 1.

Ordinary sequence and characteristics of mixing, casting and compaction used for creation of the standardized mortars were utilized [25]. From one batch of the mix (see Table 1), three prism specimens (40 mm × 40 mm × 160 mm) were cast. The curing process was divided into two stages. During the first stage, the specimens were kept in steel molds covered with glass sheets for 24 h. During the second stage, the specimens were put into a water tank for the following 27 days. The temperature of air and water during both stages of curing was constant and equal to +20 °C ± 1 °C. All cast specimens were tested after 28 days of curing.

After 28 days of curing, the cement mortar specimens (with no spatial reinforcement) were characterized by a density of 2261 kg/m^3^ and compressive strength and flexural strength equal to 45.5 MPa and 7.0 MPa, respectively. Other properties of the cement in question are thoroughly described in previous publications [26,27].

Acrylonitrile-co-butadiene-co-styrene (ABS) was chosen as a filament [28,29] for 3D printing of reinforcing elements. ABS proved to be effective as a material for the creation of non-conventional 3D printed reinforcement [16]. Key properties of the ABS filament given by the producer are presented in Table 2. The reasoning why the ABS filament was chosen and its properties were thoroughly described in a previous publication [19].

**Figure 1:**
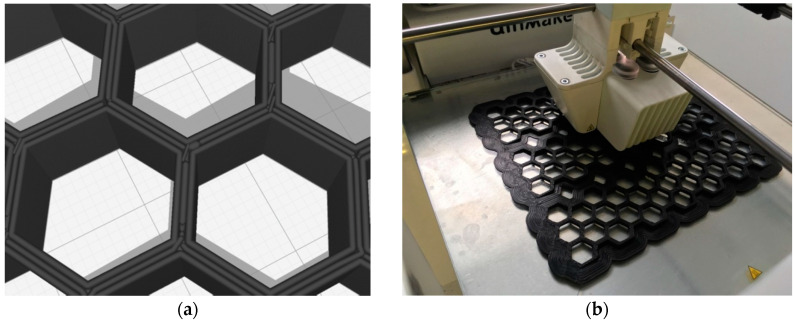
(**a**) The three-layered structure of printed hexagon walls. (**b**) A set of three spatial reinforcing elements being 3D printed (photos by T. Szatkiewicz).

The utilized 3D printing process was designed carefully to maintain three layers in every printed hexagon wall. The thickness of the walls was varied; thus, the thickness of the three layers was adjusted accordingly. Two sizes (0.6 mm and 0.8 mm) of the print cores were used. Both external layers and an infill layer of all walls were printed as solids to guarantee the best possible strength in any direction. The average speed of printing was equal to 25 mm/s. The adopted three-layered structure of printed hexagon walls is presented in Figure 1a.

For the preparation of the spatial reinforcing elements, a commercially available 3D printer was utilized. The size of the printed reinforcement, and subsequently, the size of the tested specimens, was limited by the available working space of the printer (197 mm × 215 mm). During one run of the printer, three reinforcing elements of the same geometry were created (see Figure 1b). The elements were put into the steel mold where overlay polymer helped to keep them in place at the bottom of the mold during casting (two layers) and compaction of cement mortar. The compaction was conducted using an ordinary jolting table with a drop of 15 mm. Each cast layer was compacted by 60 jolts.

**Figure 2:**
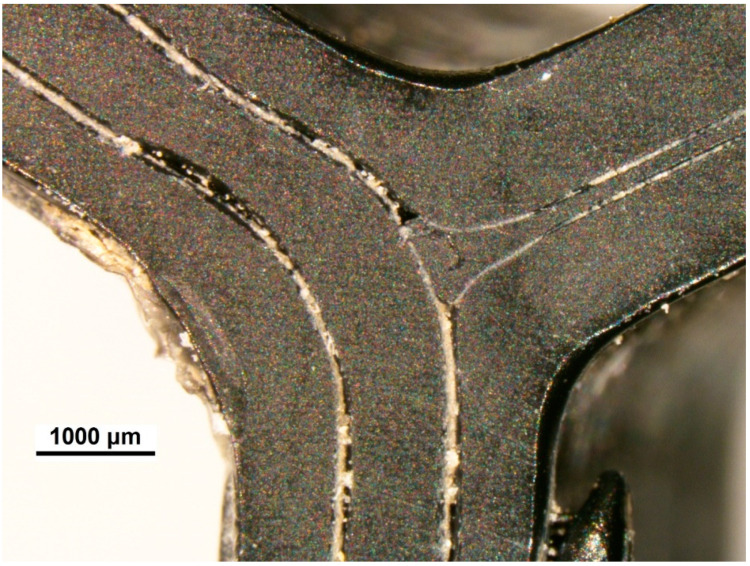
Microscopic view of three-layered printed hexagon walls.

3D printed spatial reinforcing elements were analyzed using the optic microscope. In Figure 2, one can see a wall of a hexagon consisting of three printed layers. The structure of a wall is not homogenized. Borders of specific layers are clearly visible with small crevices separating them.

**Figure 3:**
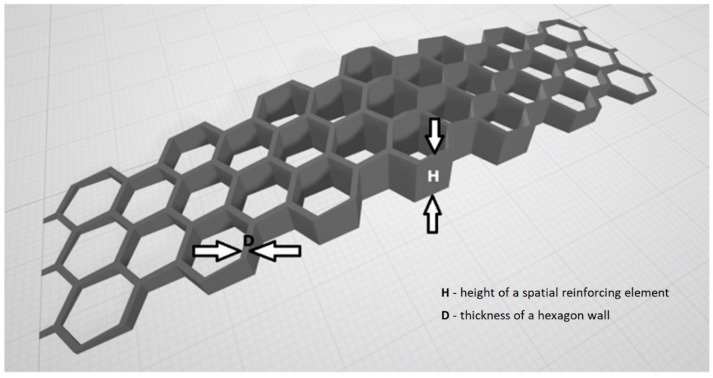
General view of the designed spatial reinforcing element.

An orthogonal experiment design was adopted for choosing types of spatial reinforcement to be tested during the research program [30]. The selection of the design of experiment was based on a previous research experience with non-conventionally reinforced concrete elements [31]. It was decided that spatial reinforcing element will have a triangular cross-section along the length of a beam. Thus, the maximum height of the reinforcing element was located in the middle of the span. The size of hexagons and their spacing was constant for all 3D printed reinforcing elements. There were only two variables: the height (*H*) of a spatial reinforcing element and the thickness (*D*) of a hexagon wall. The height was equal to 5 mm, 10 mm, 15 mm and 20 mm. The thickness of the wall was equal to 1 mm, 1.33 mm, 1.66 mm and 2 mm. The general view of the designed spatial reinforcing element with marked *H* and *D* is presented in Figure 3.

The adopted geometry of the spatial reinforcing elements and the three-layered structure of the hexagon walls influenced both the time of printing of an element and its mass. The time varied from 22 min to 111 min. Values of mass of all considered spatial reinforcing elements are presented in Table 3. The volume of polymer spatial reinforcement in cast element may reach the values much higher in comparison to traditional rebar reinforcement, which usually ranges from 1% to 1.5%. In the cases of spatial elements in question, the volume of 3D printed elements reaches the value of 2.8% in the case of the element characterized by *H* = 20 mm and *D* = 2 mm. Both the density of the used polymer and the volume of the 3D printed spatial reinforcement influence the weight of the cast element.

The tests were conducted in a random order to guarantee the objectivity. All achieved results were statistically assessed with the help of the Dixon’s *Q* test [32]. Only results assessed as bearing no gross error proceeded further for computation. A dedicated scientific software was utilized to create graphic interpretations of the 3D plots of second-degree equations.

All prepared mortar specimens were measured and weighed before conducting any other tests. Calculated density of hardened composites was considered as a basic but efficient quality and uniformity check of the casting, compacting and hardening processes [33]. Subsequently, specimens were used for the flexural strength test. Two ordinary supports (span *l* = 100 mm) and one central loading point setup were utilized with loading force (*F*) increasing at speed of 50 N/s. During the test, the midspan deflection (*d*) was recorded every 0.02 s. Due to expected variable quasi-plastic characteristics of different specimens, the flexural test was conducted until the deflection of 3 mm was achieved. At this point, all specimens were considered as ultimately destroyed. Collected data was used to create loading–deflection charts. The special interest was put on four points of loading–deflection characteristics of each element. These points were as follows: the limit of proportionality (first peak of the loading force named 1p), maximum loading force (*F_max_*), deflection equal to l/150 (p066) and deflection equal to l/75 (p133). The points were chosen to allow comparison with traditional reinforced concrete structures [34], ferrocement [35] and steel fiber-reinforced concrete (SFRC) [36]. Additionally, compressive strength of mortar with no reinforcement was tested.

## 3. Results and Discussion

**Figure 4:**
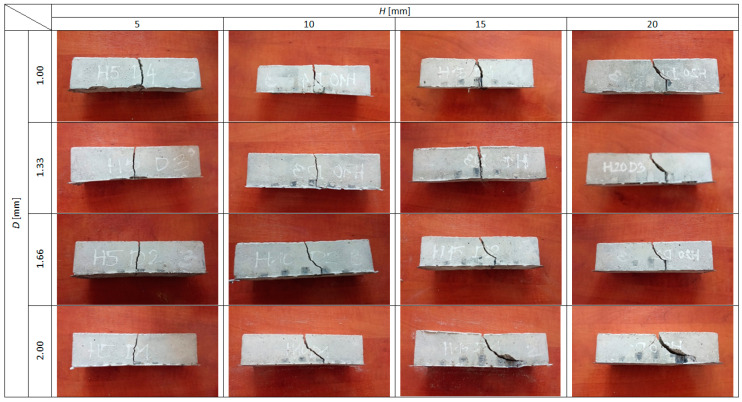
Cracking patterns of ultimately destroyed prism specimens.

**Figure 5:**
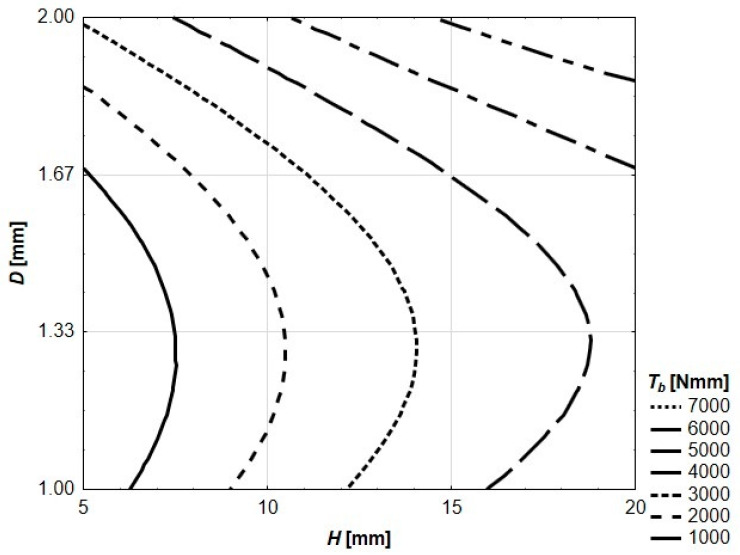
Flexural toughness of tested beams for maximum loading force.

Density, compressive strength and flexural strength of the mortar were equal to 2363 kg/m^3^, 43.3 MPa and 8.0 MPa, respectively. These values were serving as a reference point for all other mechanical characteristics of reinforced beams. Behavior of the beams during the flexural strength test was registered both in digital (force–deflection relations) and graphical (photographs) ways. The photographs showing cracking patterns of ultimately destroyed beams are presented in Figure 4. Due to quasi-plastic behavior of all tested beams (which in many ways resembles SFRC or ferrocement), the traditional approach to achieved results was disabled. On the other hand, recorded deflections at the ultimate destruction were much higher than in the case of SFRC or ferrocement, and utilization of standards referring to these cement composites was also impossible. The authors decided to use four key points to analyze flexural behavior of the tested beams. For each of four key points, the area under the curve was calculated. The calculated areas represent the amount of energy needed to get to the certain point on the load–deflection curve during the flexural strength test. The idea of using these values for further analysis was sourced from the Japanese standard JSCE-SF4 [37] describing the flexural testing method of SFRC. Similar to JSCE-SF4, the authors decided to use term flexural toughness (*Tb*) to describe these values of dissipated energy. In Figure 5, using the height (*H*) of the spatial reinforcing element and the thickness (*D*) of a hexagon wall as independent input variables, the contour plot of *Tb* for *F_max_* is presented.

The value of *T_b_* for *F_max_* ranges from 244 Nmm (for *H* = 5 mm and *d* = 1.00 mm) to 7049 Nmm (for *H* = 20 mm and *d* = 2.00 mm). The value of *T_b_* for *F_max_* is increasing alongside both the growing thickness of walls and the height of the spatial reinforcing element. The contour graph presented in Figure 4 is described by Equation (1). Both the equation and the contour graph were generated using STATISTICA software. All other contour graphs in this paper (and equations describing them) were created in the same way.

(1) *T_b_*(*F_max_*) = 7321.4 + 520.0 *H* − 14994.7 *D* − 8.3 *H*^2^ − 27.6 *H D* + 5916.8 *D*^2^

**Figure 6:**
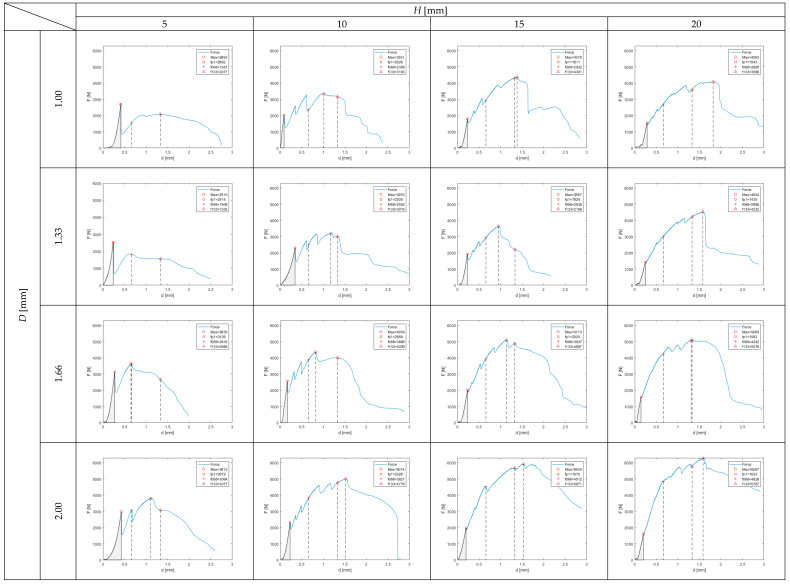
Load (*F*)–deflection (*d*) relations for tested prism specimens with marked four characteristic points (values of load achieved for each key point are listed [N]).

**Figure 7:**
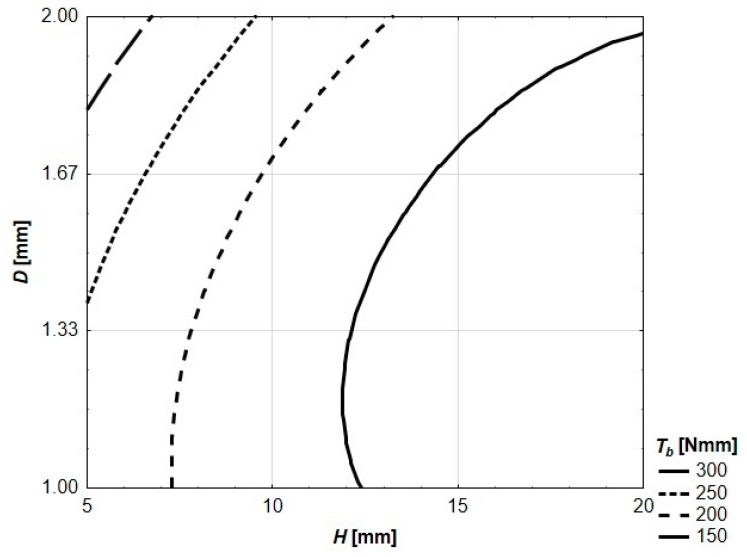
Flexural toughness of tested prism specimens for the first peak of loading force.

One should notice that the value of *F_max_* for an ordinary mortar prism specimen without any reinforcement was equal to 3413 N. It means that four reinforced beams (with spatial reinforcing elements: *H* = 5 mm and *D* = 1.00 mm, *H* = 5 mm and *D* = 1.33 mm, *H* = 10 mm and *D* = 1.00 mm, *H* = 10 mm and *D* = 1.33 mm) were characterized by lower maximum loading force than the pure mortar. The achieved values of *F_max_* were equal to 79%, 74%, 98% and 94% of the characteristics of pure mortar, respectively. On the other hand, the mortar beam reinforced with the spatial element characterized by *H* = 20 mm and *D* = 2.00 mm achieved a value of *F_max_* which is equal to 184% of the characteristics of pure mortar. In the cases of all specimens but two, the maximum loading force was achieved after reaching the first peak (see Figure 6). The behavior of the specimens up to the first peak reflects the elastic behavior of ordinary mortar (or any other brittle cement-based material). In the cases of the tested specimens, the loading–deflection relation is not as proportional as in the case of ordinary mortar (especially in the cases of specimens with reinforcement with *H* = 5 mm), but the resemblance is good enough to proceed with the further analysis. In Figure 7, the *T_b_* for 1p is presented. The contour graph is described by Equation (2). This area is marked grey in all plots in Figure 6. The highest value of dissipated energy (equal to 373 Nmm) was achieved by the beam reinforced by the spatial element of *H* = 5 mm and *D* = 2.00 mm.

(2) *T_b_*(1p) = 398.6 − 16.8 *H −* 168.4 *D* + 0.6 *H*^2^ − 5.7 *H D* + 99.8 *D*^2^

**Figure 8:**
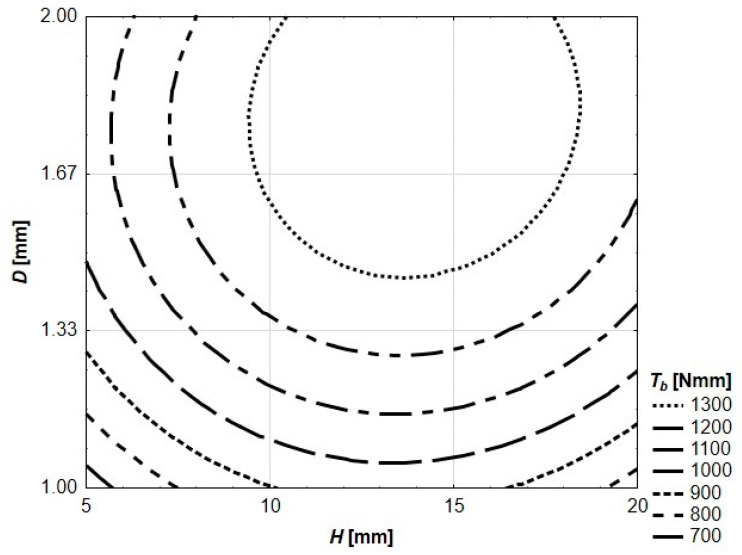
Flexural toughness of tested prism specimens for *l*/150.

**Figure 9:**
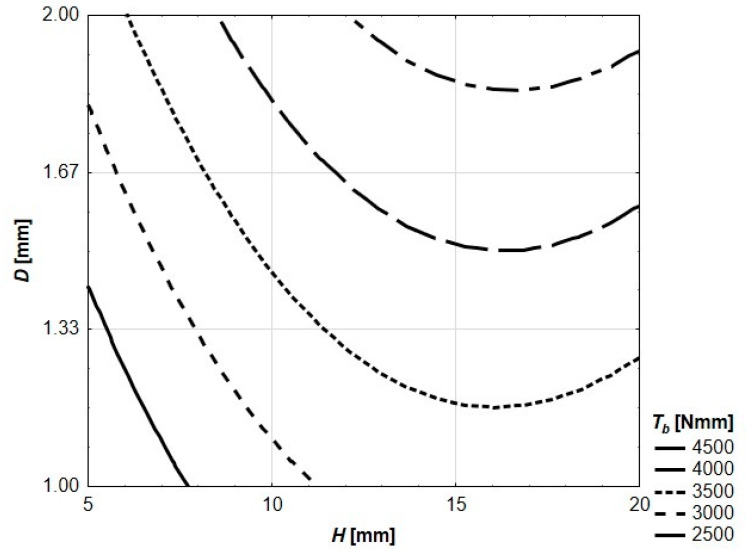
Flexural toughness of tested prism specimens for *l*/75.

In Figure 8 and Figure 9, flexural toughness for deflections *l*/150 and *l*/75 was presented, respectively. The value of *l*/150 was chosen for the analysis due to the fact that, in multiple national and international standards, such a deflection of a structural element is considered as a serviceability limit state (SLS) or ultimate limit state (ULS) [38]. The deflection of *l*/75 represents twice the deflection. In the cases of the reinforced specimens in question, only in three cases (reinforcing elements: *H* = 5 mm and *D* = 1.00 mm, *H* = 5 mm and *D* = 1.33 mm, *H* = 5 mm and *D* = 1.66 mm) did both deflections appear after reaching *F_max_*. In the cases of seven specimens, *F_max_* is located between *l*/150 and *l*/75, and in the cases of six prisms, both deflections are located before reaching *F_max_* (see Figure 6). These varied quasi-plastic characteristics of tested specimens are also visible in different crack patterns (see Figure 4). The specimens with low reinforcing elements (*H* = 5 mm) have one vertical crack in the middle of the span. When the reinforcing element is getting higher and thicker, the main crack is more and more curved, and some secondary cracks appear. In the case of the most reinforced specimen (reinforcing element *H* = 20 mm and *d* = 2.00 mm), the main crack is almost horizontal at its end with the secondary crack clearly visible and mirroring in shape the main one.

The contour graph of flexural toughness of tested prism specimens for deflection of 0.66 mm (*l*/150) presented in Figure 8 is described by Equation (3).

(3) *T_b_*(p066) = −1540.2 + 105.4 *H* + 2443.4 *D* − 4.2 *H*^2^ + 6.4 *H D* − 705.8 *D*^2^

The contour graph of flexural toughness of tested prism specimens for deflection of 1.33 mm (*l*/75) presented in Figure 9 is described by Equation (4).

(4) *T_b_*(p133) = −993.3 + 337.4 *H* + 1529.7 *D* − 11.1 *H*^2^ + 15.6 *H D* − 97.7 *D*^2^

Achieved flexural characteristics of the tested prism specimens prove that it is possible to efficiently reinforce concrete by spatial 3D printed polymer elements. The utilization of such elements enables shaping properties of cement composites at a whole new level of influencing their elastic and quasi-plastic properties. In the majority of tested specimens, their enhanced properties were seen after reaching the first peak of the loading force. On the other hand, the first peak of the loading force can be associated with the first crack of the brittle cement mortar and beginning of the full work of a spatial 3D printed plastic element. Large amount of energy needed to ultimately destroy the elements (after reaching their SLS) prone this reinforcing technology for special applications where such behaviour is needed. All kinds of structures exposed to possible impacts, explosions, shockwaves, earthquakes, etc. are coming to mind as the first possible application area. One should also keep in mind the fact that polymer filament for a 3D printer can be produced from waste polymers [39,40]. Using large amounts of recycled polymer filament for printing spatial reinforcement would create a new quality in both construction industry and environment protection. Currently existing 3D printers were developed to print small but very precise polymer elements. New kinds of 3D printers should be developed for civil engineering applications where size would be the priority, not the smoothness of the printed polymer surfaces. To fully justify the structural practicality of 3D printed spatial plastic reinforcement, some additional research should be done. Future studies should cover: comparison of structural elements reinforced by 3D printed polymer elements versus steel rebars and thorough study about “composite behavior” of mortar and 3D printed polymer reinforcement (taking into account what happens to the “interface” between two materials during thermal expansion, how significant is adhesion, etc.).

## 4. Conclusions

The conducted research program allows us to draw the following conclusions:

- It is possible to efficiently reinforce cement mortar by spatial 3D printed polymer elements.
- The shape and size of a spatial 3D printed element influence in a very wide range the flexural behavior of a prism specimen.
- Harnessing existing standards dedicated for reinforced concrete, fiber-reinforced concrete and ferrocement for testing and analysis of mortar prism specimen with spatial 3D printed plastic-reinforced elements is not feasible.
- Some elements of existing standards (e.g., flexural toughness) can be adopted for the testing and analysis of mortar prism specimens with spatial 3D printed polymer reinforcing elements.
- The shape and size of the 3D printed polymer reinforcing elements should be further developed to optimize their efficiency.
- Tests on larger specimens and full-scale structural elements (e.g., beams, columns) should be conducted.

## Figures and Tables

**Table 1:** Mixture composition for one batch.

Ingredient	Amount [g]	Density [g/cm^3^]	Volume [cm^3^]
Standardized sand	1350	2.65	509.4
Portland cement	450	3.10	145.1
Tap water	225	1.00	225.0

**Table 2:** Key properties of the ABS filament used in this study.

Density [kg/cm^3^]	Melting Point [°C]		Diameter [mm]		Thermal Decomposition [°C]	
1100	+225		2.85		>+280	
Tensile modulus [MPa]	Tensile stress at yield [MPa]	Tensile stress at break [MPa]	Elongation at yield [%]	Elongation at break [%]	Flexural strength [MPa]	Flexural modulus [MPa]
1681.5	39.0	33.9	3.5	4.8	70.5	2070.0

**Table 3:** Mass of spatial reinforcing elements.

		***H* [mm]**			
		5	10	15	20
***D* [mm]**	1.00	1.25	2.25	3.2	4.17
	1.33	1.42	3.55	3.71	4.87
	1.66	2.07	3.60	5.09	6.60
	2.00	2.35	4.18	5.98	7.80
